# Coupled versus decoupled visuomotor feedback: Differential frontoparietal activity during curved reach planning on simultaneous functional near‐infrared spectroscopy and electroencephalography

**DOI:** 10.1002/brb3.2681

**Published:** 2022-06-14

**Authors:** Duc Trung Le, Hiroki Ogawa, Masato Tsuyuhara, Kazuki Watanabe, Tatsunori Watanabe, Ryosuke Ochi, Hisao Nishijo, Masahito Mihara, Naoto Fujita, Susumu Urakawa

**Affiliations:** ^1^ Department of Musculoskeletal Functional Research and Regeneration, Graduate School of Biomedical and Health Sciences Hiroshima University Hiroshima Japan; ^2^ Department of Sensorimotor Neuroscience, Graduate School of Biomedical and Health Sciences Hiroshima University Hiroshima Japan; ^3^ Department of System Emotional Science, Graduate School of Medicine and Pharmaceutical Science University of Toyama Toyama Japan; ^4^ Research Center for Idling Brain Science (RCIBS) University of Toyama Toyama Japan; ^5^ Department of Neurology Kawasaki Medical School Okayama Japan

**Keywords:** EEG, feedback, fNIRS, frontoparietal cortex, reach planning, visuomotor transformation

## Abstract

**Introduction:**

Interacting with the environment requires the planning and execution of reach‐to‐target movements along given reach trajectory paths. Human neural mechanisms for the motor planning of linear, or point‐to‐point, reaching movements are relatively well studied. However, the corresponding representations for curved and more complex reaching movements require further investigation. Additionally, the visual and proprioceptive feedback of hand positioning can be spatially and sequentially coupled in alignment (e.g., directly reaching for an object), termed coupled visuomotor feedback, or spatially decoupled (e.g., dragging the computer mouse forward to move the cursor upward), termed decoupled visuomotor feedback. During reach planning, visuomotor processing routes may differ across feedback types.

**Methods:**

We investigated the involvement of the frontoparietal regions, including the superior parietal lobule (SPL), dorsal premotor cortex (PMd), and dorsolateral prefrontal cortex (dlPFC), in curved reach planning under different feedback conditions. Participants engaged in two delayed‐response reaching tasks with identical starting and target position sets but different reach trajectory paths (linear or curved) under two feedback conditions (coupled or decoupled). Neural responses in frontoparietal regions were analyzed using a combination of functional near‐infrared spectroscopy and electroencephalography.

**Results:**

The results revealed that, regarding the cue period, curved reach planning had a higher hemodynamic response in the left SPL and bilateral PMd and a smaller high‐beta power in the left parietal regions than linear reach planning. Regarding the delay period, higher hemodynamic responses during curved reach planning were observed in the right dlPFC for decoupled feedback than those for coupled feedback.

**Conclusion:**

These findings suggest the crucial involvement of both SPL and PMd activities in trajectory‐path processing for curved reach planning. Moreover, the dlPFC may be especially involved in the planning of curved reaching movements under decoupled feedback conditions. Thus, this study provides insight into the neural mechanisms underlying reaching function via different feedback conditions.

## Graphical abstract

Graphical Abstract:

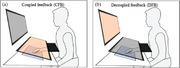

## 1 INTRODUCTION

Stroke is the second most common cause of mortality and the third most common cause of disability worldwide (Lozano et al., 2012). Despite considerable progress in managing acute stroke, many stroke survivors experience various functional deficits that can diminish their ability to perform daily tasks. Upper‐limb motor dysfunction is the most common symptom that occurs following stroke, with a prevalence of almost 77% in patients reporting symptoms (Lawrence et al., 2001). The ability to reach for objects, which requires spatiotemporal coordination of the upper‐limb neuromuscular system, is necessary for a wide variety of activities of daily living (Kilbreath & Heard, 2005). Therefore, regaining reaching function is an essential goal during stroke rehabilitation. Evaluating the effectiveness of upper‐limb rehabilitation interventions requires a thorough knowledge of the neural mechanisms underpinning reaching function.

Daily living tasks involve the planning and executing of target‐directed reaching movements along given trajectory paths. Linear movement paths, the simplest form of reach trajectory, are defined by the difference vector between the hand and target positions (e.g., point‐to‐point movements). Curved reach trajectories are determined not only using the target and hand positions but also via the specific trajectory path along which the hand is oriented (e.g., obstacle circumvention, drawing, or tool use movements). To plan a target‐directed reaching movement, the brain must conduct a visuomotor transformation that converts the relevant sensory inputs into motor commands (Buneo & Soechting, 2009; Sober & Sabes, 2005). Particularly, the brain network evaluates sensory inputs signaling the target and initial hand positions (visual and/or proprioceptive feedback) to encode the impending reach trajectory‐path as neuronal representations. Based on these representations, reach‐related limb kinematics are estimated before the reach is executed. It is well established that the superior parietal lobule (SPL) and dorsal premotor cortex (PMd), which belong to the frontoparietal cortex system, play critical roles in the visuomotor transformation of target‐directed reaching movements (Beurze et al., 2010). The properties of upcoming reaching movements are spatially and kinematically represented in the planning activities of the SPL (Gallivan et al., 2011; Hawkins et al., 2013; Pilacinski et al., 2018) and PMd (Ochiai et al., 2002; Pearce & Moran, 2012; Pesaran et al., 2006) regions.

The processing of upcoming curved reach trajectories is purportedly more computationally demanding than that of linear trajectories (Torres et al., 2013; Wong et al., 2016a). A static trajectory‐path representation, such as a visual picture of the hand‐target vector, appears to be optimal for linear reach planning. Such a representation is likely inadequate for planning curved reach movements that are more spatially complicated than linear reaches (Torres et al., 2013; Wong et al., 2016a). Specifically, the representations for an upcoming curved reach movement have been suggested to include the trajectory path in motion and general knowledge of how to execute that trajectory (e.g., curved, sinusoidal, or complex arm movements). Several studies have shown that the frontoparietal regions may contribute to the processing of computationally demanding representations required for curved reach planning (Hauschild et al., 2012; Pilacinski & Lindner, 2019; Torres et al., 2013). Evidence in nonhuman primates has indicated that the neuronal representations of an impending curved reach trajectory may be encoded in the SPL region (Hauschild et al., 2012; Torres et al., 2013). Meanwhile, few human studies using functional magnetic resonance imaging (fMRI) have explored the brain correlates of curved reach planning (Pilacinski & Lindner, 2019; Wong et al., 2019). Among them, one fMRI study directly investigated motor planning of curved reaching movements (Pilacinski & Lindner, 2019). This study demonstrated that PMd activity may play a major role in processing essential aspects of upcoming curved movement trajectories. With its superior spatial resolution, fMRI has become the gold standard of brain imaging through its measurement of the blood‐oxygen‐level‐dependent (BOLD) response. However, fMRI experimental environments are less natural due to their confined space, loud scanner noise, and the requirement to lay supine during scanning (Uddin et al., 2010). These factors can cause a differentiation in cognitive demand and visuospatial orientation relative to real‐world environments. Moreover, due to its susceptibility to motion artifacts, fMRI‐based reaching tasks are limited to wrist and finger movements (Koehler et al., 2012), which may involve a motor control strategy that is different from natural reaching movements, which predominantly involve elbow‐centered excursions. Therefore, the human neural mechanisms of curved reach planning require further corroboration and analysis using less restrictive neuroimaging modalities and experimental settings that better reflect the real world.

Functional near‐infrared spectroscopy (fNIRS) has emerged as a noninvasive, practical imaging tool that measures hemodynamic changes at the cortical level. fNIRS signals have been shown to be associated with cortical activation (Okamoto et al., 2004) and BOLD signals (Cui et al., 2011), indicating the feasibility of fNIRS analysis for detecting human brain activity. Since this modality has no restrictions on participants and has high tolerance against motion artifacts, fNIRS provides an ideal technique for researching various demanding motor tasks in unconstrained settings, including reaching movements (Goto et al., 2011; Ishikuro et al., 2014), walking (Mihara et al., 2007), and social interactions (Urakawa et al., 2015). In addition to the neuroimaging modalities already mentioned, electroencephalography (EEG) is one of the most commonly applied methods for detecting brain physiological activity through electrodes placed on a participant's scalp. As a portable and noninvasive functional neuroimaging modality, EEG has been used to investigate various aspects of motor planning of visually guided movements (Tzagarakis et al., 2010, 2021). Visuomotor transformation for impending arm movements is reportedly reflected by changes in the frontal and parietal beta power of EEG (Liebrand et al., 2018; Perfetti et al., 2011; Tombini et al., 2009; Wheaton et al., 2008). Moreover, oscillatory activity at beta frequencies contains the information needed to decode future arm‐movement trajectories (Korik et al., 2018). The utility of combined fNIRS‐EEG recordings has been demonstrated in various visuomotor tasks involving target‐directed movements (Chiarelli et al., 2017; Zama et al., 2019). This multimodal brain imaging approach enables an assessment of cortical activation from both a hemodynamic and neurophysiological perspective without interfering with each other's signals (Biallas et al., 2012). Hence, the simultaneous measurement of fNIRS and EEG is likely an optimal neuroimaging approach for studying visuomotor transformation for reaching movements.

Reach planning processes are also influenced by sensory feedback from the hand (Crocher et al., 2019). Visual and proprioceptive feedback from hand positioning is generally perceived in a spatially coupled manner (e.g., reaching for an object with the hand), which is termed coupled visuomotor feedback. However, this feedback can also be spatially decoupled or mapped on distinct spatial coordinates, as is usually experienced with virtual hand representations under computer‐based conditions (e.g., dragging a computer mouse horizontally to move the cursor vertically), which is termed decoupled visuomotor feedback. To account for the vision‐proprioception decoupling of hand positioning, reach planning under decoupled feedback may require extra processing to transform motor plans from vision to hand‐centered coordinates (Bo et al., 2006; Dalecki et al., 2019a; Veilleux & Proteau, 2011). This process of coordinate transformation may involve encoding and incorporating transformational rules (e.g., dragging the mouse forward to move the cursor upward) into reaching plans (Granek & Sergio, 2015). In line with this view, evidence in nonhuman primates (Hawkins et al., 2013; Sayegh et al., 2013, 2014) has found alterations in the activity of the SPL and PMd regions associated with reach planning under decoupled feedback. Additionally, activation of the dorsolateral prefrontal cortex (dlPFC) and the rostral prefrontal cortex (rPFC)—which govern rule‐based behaviors (Badre & Nee, 2018; Hoshi, 2013; Miller & Cohen, 2001)—has been found to be modulated during planning for visuomotor coordination in decoupled feedback conditions (Granek et al., 2010). However, previous studies conducted under decoupled feedback conditions have been limited to linear reaching movements. Regarding reach planning under decoupled feedback, curved reach trajectories are more spatially complex and seem more computationally demanding when it comes to the processing of coordinate transformations compared to linear trajectories. It remains unclear how cortical activities change during curved reach planning in decoupled relative to coupled feedback conditions and how they compare to those during linear reach planning. The use of decoupled feedback conditions in reach rehabilitation is becoming more common (Crocher et al., 2019), necessitating a better knowledge of the underlying brain processes.

In the present study, we used a simultaneous fNIRS‐EEG approach to investigate frontoparietal involvement in planning curved reaching movements under coupled and decoupled visuomotor feedback conditions. Using two types of reach trajectory paths (linear and curved) and two types of visuomotor feedback conditions (coupled and decoupled), we examined how these different experimental conditions affected the frontoparietal activity associated with the processes of trajectory representation and feedback‐related coordinate transformation during reach planning. The participants’ neural activities were measured by fNIRS‐EEG recordings while they performed delayed‐response reaching tasks. Research on motor planning is best investigated using delayed‐response paradigms (Hikosaka & Wurtz, 1983; Sober & Sabes, 2005). Such paradigms can aid in the division of planning processes into two stages: including cue periods and their following delay periods. The reach‐planning activity during the cue period may involve trajectory‐related processing (Pilacinski et al., 2018), whereas the process of coordinate transformation may prevail during the delay period (Gorbet & Sergio, 2016). Consequently, we hypothesized that comparing two reaching tasks would reveal trajectory‐path processing for curved reach planning, reflected by increased cue‐period activation in the SPL and PMd regions. We further hypothesized that contrasts between these two feedback conditions would elucidate processes that incorporate transformational rules into reaching plans, thereby modulating delay‐period activity in the frontoparietal regions. This study aimed to contribute to a better understanding of the brain processes that underlie curved reach planning. The study's findings may assist in comprehending the cortical effects of arm reaching practices, further supporting the improvement of interventions and strategies in reach rehabilitation.

## 2 METHODS

### 2.1 Participants

In total, 19 healthy participants (10 women and nine men; age range: 21.0 ± 1.7 years) were enrolled in this study. Participants were right‐hand dominant, assessed using the Edinburgh Handedness Questionnaire (90.0 ± 13.3). No participant had a medical history of neurological or psychiatric disorders or any orthopedic injuries that could have reduced upper‐limb sensorimotor function. Participants were instructed to avoid consuming any alcohol‐ or caffeine‐containing substances for at least 12 h before the experiments. Participants were informed about the purpose of the research and subsequently provided written informed consent prior to participating in the experiment. All procedures complied with the Declaration of Helsinki and the United States Code of Federal Regulations for the Protection of Human Participants. This study was approved by the Human Ethics Committee of Hiroshima University (No. E‐2216). Three participants were excluded from the analysis due to excessive artifactual EEG signals (*n* = 2) and technical failure with EEG recordings (n = 1). Hence, we analyzed data from 16 participants (nine women and seven men, age range: 21.0 ± 1.7 years).

### 2.2 Material and feedback conditions

**FIGURE 1:**
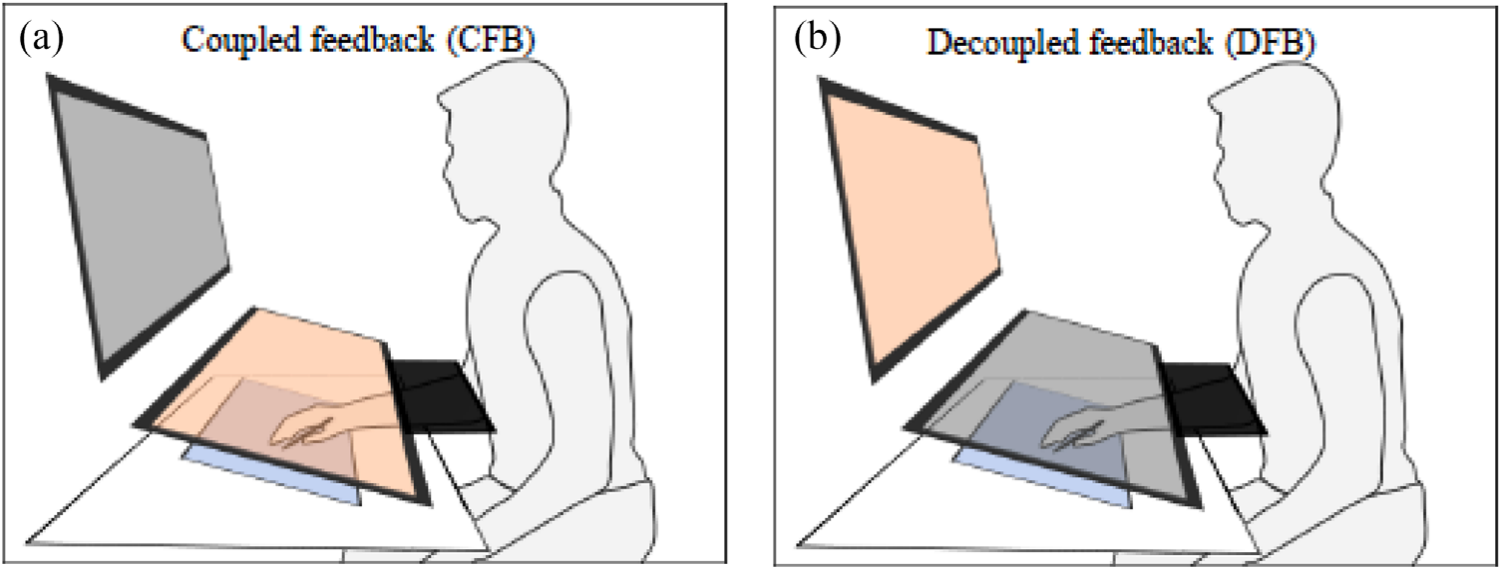
Visuomotor feedback conditions in the coupled feedback condition (a), participants manipulate the stylus while fixating on a horizontal monitor. In the decoupled feedback condition (b), participants manipulate the stylus horizontally while fixating on a vertical monitor

Participants sat on a chair at a table with vertically stacked dual monitors (23″ Flexscan EV 2316V, Eizo, Japan; 24″ V243, HP, USA; set up in the vertical and horizontal planes, respectively). The distances between a participant's nasion and the center of the monitors were equally adjusted to between 45 and 50 cm (Figure 1). A digital tablet (active area size: 8.5″ by 5.3″ Intuos, Wacom, Japan) paired with a stylus (Intuos, Wacom, Japan) was horizontally fixed on the table and angled at 10°. Elbow and chin rests were used to limit excess arm and head movements during the experiments. By manipulating the stylus using the right hand, which was constrained by a wrist splint, participants performed reach‐to‐target movements displayed on one of the dual monitors with target cues and a cursor matching the position of the stylus on the tablet. The cursor gain, or the ratio between the stylus and cursor motions, was set to 1.0 (e.g., dragging the stylus 10 cm on the tablet moved the cursor 10 cm on the monitor).

Participants engaged in two separate sessions corresponding to two feedback conditions: a coupled feedback condition (CFB) and a decoupled feedback condition (DFB). In the CFB (Figure 1a), participants manipulated the stylus while fixating on a horizontal monitor set up at a 10° angle from the table. The horizontal monitor was adjusted so that the display was parallel to the tablet surface, providing hand feedback visually aligned to actual hand motion. In the DFB (Figure 1b), participants manipulated the stylus horizontally while fixating on the vertical monitor. In the former condition, the visual and somatosensory senses of hand motions were spatially coupled, whereas spatial coupling was dissociated in the latter condition. A black cover was used to block the view of the participant's hand, ensuring that the visual stimuli in the two conditions were relatively identical. While the visual information across the two feedback settings was comparable in this investigation, the association between vision and proprioception in hand positioning was more spatially dissociated in DFB than it was in CFB (Bo et al., 2006; Dalecki et al., 2019b; Veilleux & Proteau, 2011). To account for the decoupling of vision and proprioception in the hand positioning in DFB, reach planning may necessitate additional processing to convert motor plans from vision to hand‐centered coordinates than in CFB (Granek & Sergio, 2015). Therefore, DFB and CFB could provide a good comparison to investigate the underlying neural mechanisms of coordinate transformation.

There were two reasons we used a computer‐based setting instead of a direct‐feedback setting with visible hand movements for the coupled feedback condition. First, coupled feedback conditions using natural or computer‐based settings may demonstrably share a relatively similar processing route for visuomotor transformation (Bo et al., 2006; Veilleux & Proteau, 2011). Second, providing virtual representations of the hand and target positions would normalize the amount of visual information perceived under both conditions. A direct view of hand action involves a natural tendency to link eye and hand movements together. However, when a virtual representation replaces the hand, this eye‐hand linkage is inhibited, seemingly involving additional frontoparietal processing (Sayegh et al., 2013). Thus, using computer‐based settings, a comparison of decoupled and coupled feedback conditions may control the effect of such inhibition processes, further revealing the cortical activity associated with the process of coordinate transformation.

### 2.3 Task procedure

**FIGURE 2:**
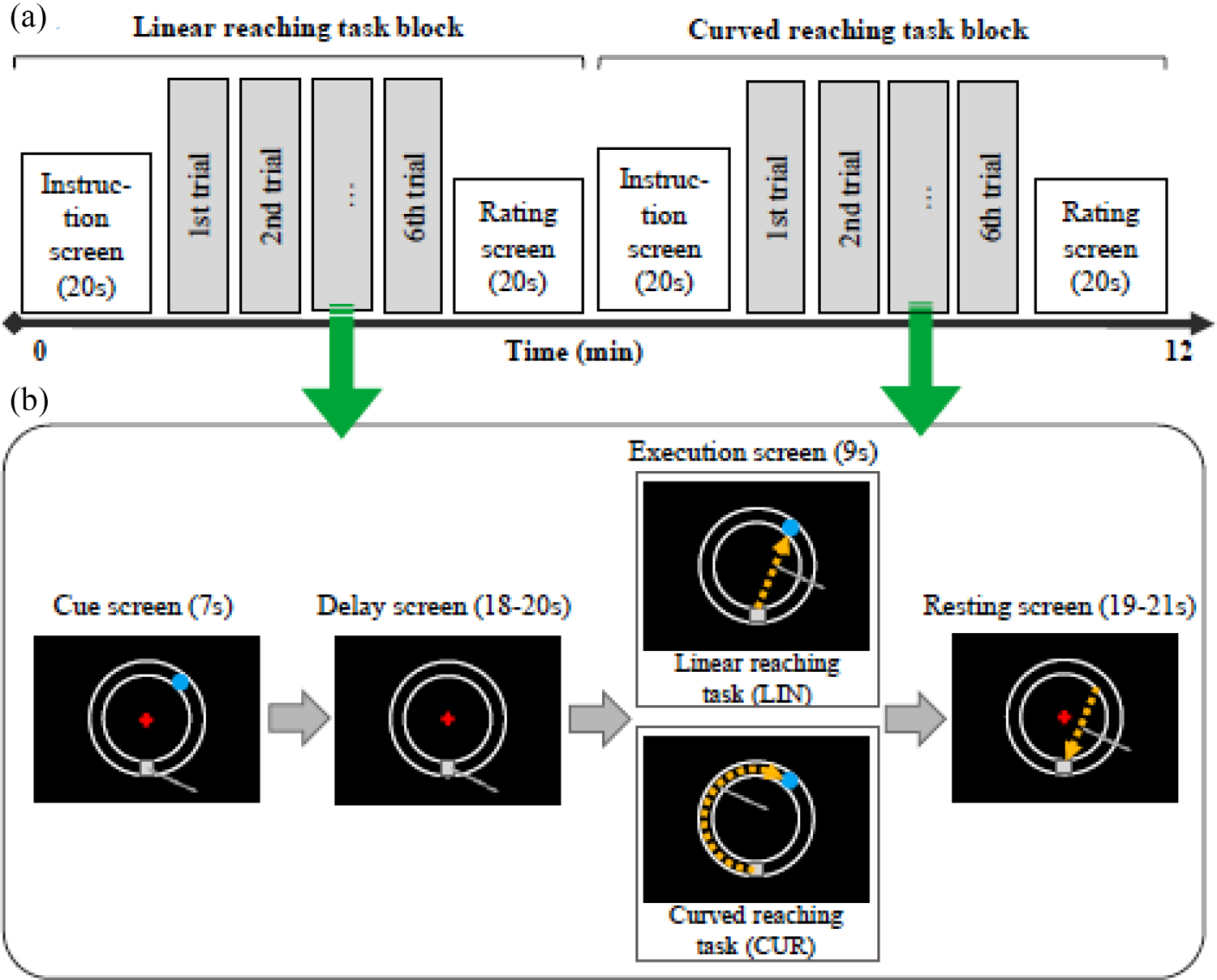
Reaching task design. (a) Simplified scheme of an experimental session (coupled or decoupled feedback condition) with two task blocks. Each task block begins with a 20 s instruction screen, six consecutive task trials, and a 20 s rating screen. (b) Design of linear and curved reaching tasks. The task begins with a cue screen, during which a target is pseudo randomly presented on the left or the right side of the trajectory path at one of six predesigned locations (−90, −126, −162, and 90, 126, 162, respectively)

The experiments and stimuli were programmed using PsychoPy (PsychoPy3, University of Nottingham, UK). Each session consisted of two reaching‐task blocks, evenly divided into six consecutive task trials of either the linear reaching task (LIN) or the curved reaching task (CUR) (Figure 2a). The order of the conditions was pseudorandomized across participants.

We employed a 2 × 2 factorial design (reaching tasks × feedback conditions) with a delayed‐response paradigm to investigate the neural mechanisms underlying curved reach planning in different feedback conditions. Such mechanisms are hypothetically associated with the processes of trajectory representation and feedback‐related coordinate transformation during reach planning. Delayed‐response paradigms can help separate planning processes into two relatively distinct stages, including cue periods and their subsequent delay periods. Reach planning activity during the cue period may represent trajectory‐related processing, whereas the process of coordinate transformation may occur predominantly during the delay period. Previous fMRI research on reach planning has used a variety of cue and delay period lengths (Gorbet & Sergio, 2016, 2019; Pilacinski & Lindner, 2019). Considering that the two effects in this study may prevail at different stages of planning activities, an appropriate interval setting is required for cue and delay periods. It is also known that a task‐related hemodynamic response takes several seconds to peak and does not return to baseline immediately (Cui et al., 2010; Ichikawa et al., 2014). Based on the above considerations as well as our preliminary results, we set up the task trials using a delayed‐response paradigm, with a cue screen for 7 s, a delay screen for 18−20 s, an execution screen for 9 s, and a resting screen for 19−21 s (Figure 2b).

Before participants engaged in each task block, an instruction screen (10 s) was presented to notify participants of which type of reaching task was required for the upcoming six trials. Afterward, a preparation screen (20 s) showed the main experimental display with a visual angle of less than 15° for the active area. This main display comprised a circular trajectory path formed by two concentric circles (radius: 63.5 and 50.8 mm), a cross in the middle of the screen, and a rectangular starting zone placed on the bottom of the path where the reaching movements would be initiated. Participants were initially asked to move a circular cursor (radius: 3.175 mm) to the starting zone and fix the cross. Each reaching trial began with a cue screen for 7 s, during which a circular target was presented on the left or right side of the trajectory path at one of six predesigned locations (−90°, −126°, −162° and 90°, 126°, 162°). Cue screens involved planning the preinstructed reaching movement toward the target in either a straight line or a curve along the longer part of the trajectory‐path. Following the cue screen, a delay screen with the main display appeared and lasted for 18−20 s until the execution screen began. During the execution screen, participants were given 9 s to move the cursor to the precued target position as quickly and accurately as possible according to the planned movement. The trial ended with the main display for 19−21 s. The total time for the complete experiment was 35 min. Before participating in each feedback condition, participants were exposed to several control trials for familiarization. We compared two reach planning tasks with identical starting and target points but different instructed reach trajectories. The processing of the upcoming curved reach trajectory may require additional computation on the trajectory‐related representations than that required by the linear trajectory (Pilacinski & Lindner, 2019; Wong et al., 2016b). Using this study design, the contrast of CUR versus LIN would provide insight into the planning activity associated with the process of curved reach trajectory representations.

### 2.4 Behavioral performance and analysis

Behavioral outcome measures for task performance included movement error and velocity. The movement error for each trial was determined as the area (cm^2^) of the intersections between the ideal and actual movement paths. The ideal path for each trial was defined as either a straight line or a perfect curve connecting the starting zone to the targets, corresponding to the LIN and CUR, respectively. The movement velocity (cm/s) was generated by dividing the movement distance by the relevant movement duration obtained from the moment when the cursor left the starting zone until it reached the target. For the statistical analyses, movement errors and movement velocities were averaged across trials by condition and subject. Four trials (1.041%) were excluded from the analyses because of incorrect behaviors (e.g., failed or incorrect reaches, slow reaction time).

An agency rating screen was presented at the end of each task block, displaying a Likert scale with scores ranging from 1 (strongly disagree) to 9 (strongly agree). Participants were given 20 s to report how much their impressions of the cursor control were created by themselves. It is generally accepted that spatial coupling between the visual and somatosensory feedback of hand motions, which is indirectly reflected in a sense of agency (Frith et al., 2000), is likely to be decreased under decoupled versus coupled feedback conditions. The findings from agency measures would provide additional behavioral information about reach performance during different feedback conditions.

### 2.5 fNIRS‐EEG simultaneous measurement

**FIGURE 3:**
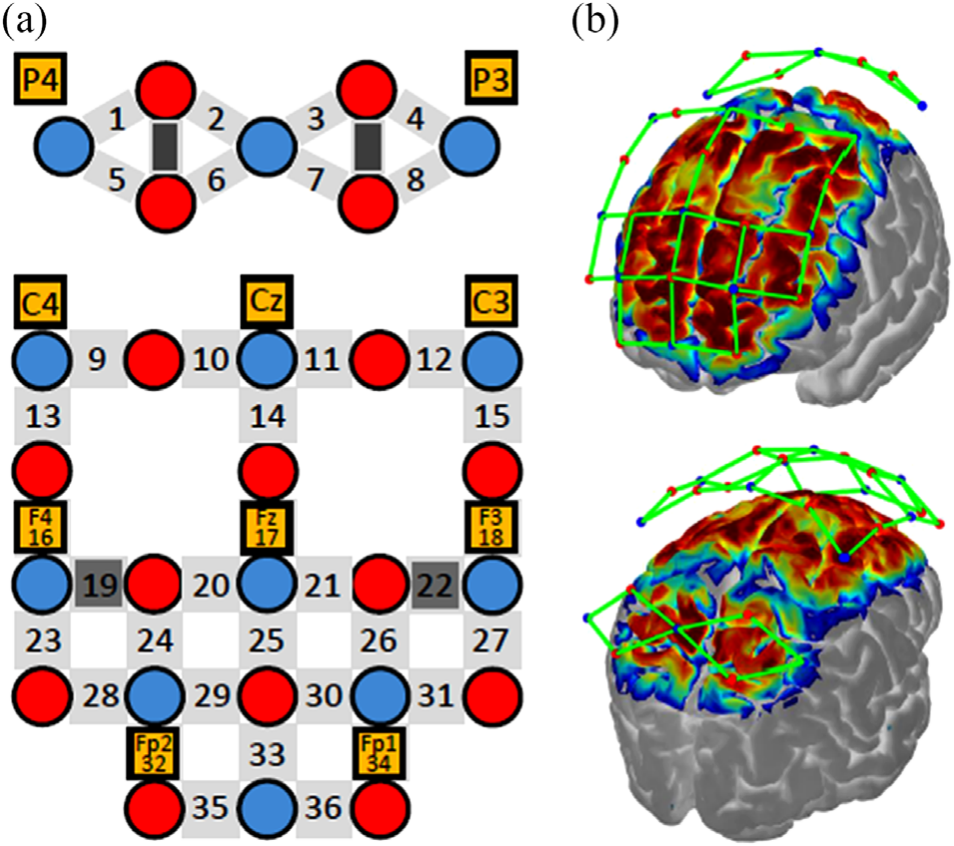
Functional near‐infrared spectroscopy‐electroencephalography (fNIRS‐EEG) cap configuration. (a) fNIRS optode and EEG electrode layout design. fNIRS sources and detectors are represented by red‐ and blue‐filled circles, respectively. Long‐ and short‐separation channels are represented by light and dark gray rectangles, respectively. EEG electrodes (orange) are placed according to the International 10–20 EEG electrode system. (b) Cortical activation mapping reveals estimated spatial information of the measurement on the surface of the brain cortex using the current fNIRS optode configuration

The fNIRS optodes and EEG electrodes were fixed to the participant's scalp using a customized fNIRS‐EEG head cap based on the International 10–20 EEG electrode system (Figure 3). fNIRS data were measured using a 32‐probe layout (16 sources and 16 detectors) that covered the frontoparietal cortical regions, including the SPL, PMd, dlPFC, and rPFC (Figure 3b). The optodes comprised 40 long‐separation channels (with a 3 cm source‐detector distance). As fNIRS signals are derived from regional cortical and scalp blood flow (Takahashi et al., 2011), we used four short‐separation channels with a 1.5 cm source‐detector distance to detect scalp hemodynamic artifacts. The signals from these short channels were utilized for data processing. EEG data were collected from 10 Ag/AgCl active electrodes (Fp1, Fp2, F3, Fz, F4, C3, Cz, C4, P3, and P4) (Figure 3a). To identify eye movement‐related artifacts in EEG signals, electrooculography (EOG) data were measured from four electrodes placed above and below the right eye and lateral to the outer canthi of the right and left eyes.

To synchronize the EEG and fNIRS data, event trigger signals were simultaneously sent to both devices using an ethernet cable. To obtain channel‐related anatomical information, a 3D digitizer (FASTRAK, Polhemus Inc., USA) was used to record the three‐dimensional position of each optical probe and five reference landmarks, including the nasion, inion, Cz, left auricular, and right auricular points. Channel locations were estimated from the coordinates of optodes and reference points using the Montreal Neurological Institute standard space coordinates.

### 2.6 fNIRS configuration and analysis

A multichannel fNIRS system (FOIRE‐3000; Shimadzu Inc., Japan) was employed to measure cortical hemodynamic activity during experiments at a sampling rate of 7.69 Hz. Specifically, the measured changes in light absorption recorded at three wavelengths (780, 805, and 830 nm) by semiconductor laser diodes were transformed into corresponding concentration changes in oxygenated hemoglobin (oxy‐Hb), deoxygenated hemoglobin (deoxy‐Hb), and total hemoglobin (total‐Hb) using the modified Lambert–Beer law (Delpy et al., 1988). These values were measured using the unit of molar concentration multiplied by the length (mM × mm). Given that changes in oxy‐Hb signal are the most sensitive indicator of changes in regional cortical blood flow and have the highest signal‐to‐noise ratio (Okamoto et al., 2004), the analysis and discussion in this study focused primarily on the changes in the oxy‐Hb concentration. For data quality control, the results of our fNIRS analysis based on deoxy‐Hb changes are provided as supporting information (Tables S1 and S2).

To anatomically label fNIRS channels, probabilistic mapping between each fNIRS channel and its corresponding Brodmann area (BA) was performed using the open‐source software package NIRS‐SPM (BISP Lab, Korea) implemented in MATLAB (MathWorks Inc., USA). Channel sets for regions of interest (ROIs) were selected based on BAs and the anatomical locations of brain areas for each participant (Table 1).

**TABLE 1:** Anatomically labeled functional near‐infrared spectroscopy (fNIRS) channel locations using Brodmann areas. The channel sets for regions of interest (ROIs) were individually adjusted for each participant based on NIRS‐SPM probabilistic mapping

Channel	BA	ROI	Abbreviation
1, 2, 5, 6	BA 7	Right superior parietal lobule	Right SPL
3, 4, 7, 8	BA 7	Left superior parietal lobule	Left SPL
3, 10, 13	BA 6	Right dorsal premotor cortex	Right PMd
11, 12, 15	BA 6	Left dorsal premotor cortex	Left PMd
19, 20, 24	BA 9, 46	Right dorsolateral prefrontal cortex	Right dlPFC
21, 22, 26	BA 9, 46	Left dorsolateral prefrontal cortex	Left dlPFC
29, 32, 35	BA 10	Right rostral prefrontal cortex	Right rPFC
30, 34, 36	BA 10	Left rostral prefrontal cortex	Left rPFC

Abbreviation: BA, Brodmann's areas.

fNIRS signals were first processed using moving standard deviations (SDs) and spline interpolation methods to detect and reduce motion artifacts. The SD of each data segment was calculated, and motion artifacts were identified based on the SD threshold. Spline interpolation was applied to the data segments containing the motion artifact. A bandpass filter with a 0.01−0.1 Hz cutoff frequency range was then used to remove concomitant systemic responses from the signal. Subsequently, we employed a method known as direct subtraction to eliminate the extracerebral hemodynamic components from the neural data. Each long channel was paired with the short channel closest to it. By subtracting the corresponding short channel signal from the long channel signal, the corrected hemodynamic response was acquired. The preceding data analyses were performed with the use of commercial fNIRS analysis software (Advanced ROI; WAWON DIGITECH, Japan). The oxy‐Hb time courses for the planning and execution periods in each channel were corrected to the baseline values, determined as the mean over −5 s to −1 s prior to the relevant period onsets. The corrected time courses were then averaged across trials and ROI‐wise channels to generate the ROI time course for each experimental condition. Based on the ROI time course, mean oxy‐Hb changes were used as an index of cortical activation and were calculated separately for the cue planning (0−7 s), delay planning (9−16 s), and execution (0−7 s) phases with the relevant period onsets set at time zero.

### 2.7 EEG configuration and analysis

A multichannel EEG system (ActiveTwo, BioSemi Inc., Netherlands) was used to record EEG data at a sampling rate of 2048 Hz with an online bandpass filter of 0.16−100 Hz. Two electrodes, a common mode sense active electrode and a driven right leg passive electrode, were used to provide a ground reference for active electrodes (see www.biosemi.com/faq/cms&drl.htm). The direct current offset voltage was maintained below 50 mV.

Offline EEG data processing and analysis were carried out using EEGLAB (Version 2019.1, Swartz Center for Computational Neuroscience, US) and built‐in MATLAB scripts. The individual data were downsampled to 512 Hz, bandpass filtered from 1 to 100 Hz, and notch filtered at 60 Hz to attenuate line noise. Muscular and eye movement‐related artifacts were identified and excluded using an independent component analysis on EEG channels. Data were then segmented into 30‐s epochs, from −5 s precue to 25 s postcue. Epochs were manually removed from the analysis if they met any of the following criteria: (1) the signal amplitudes exceeded 200 μV; (2) epochs were heavily contaminated by eye blinks, with ocular artifacts detected using the EOG data; or (3) epochs with incorrect behaviors. As a result, 12 trials (3.125%) were excluded from the analysis. Only channels in the ROIs were included in the analysis (frontal area: F3, F4; parietal area: P3, P4).

Periods for analyzing EEG phases were set up to be the same as those used in the fNIRS analysis, with the cue planning phase (0−7 s) and the delay planning phase (9−16 s). Using Welch's periodogram method, we estimated the power spectral density for each period with a 1‐s Hanning window and a 0% overlap between segments. Next, we calculated the average log‐transformed power across low‐ (13−20 Hz) and high‐beta (21−30 Hz) bands, which reflect trajectory‐path processing (Korik et al., 2018), for each ROI channel. The relative change in the frequency power index was computed separately for each epoch as planning‐related power divided by baseline‐related power, with a baseline of −5 s to −1 s, and then averaged across epochs for group statistical analysis.

### 2.8 Statistical analysis

Two‐way repeated‐measures analysis of variance (ANOVA) tests were used to examine the effects of reaching tasks (LIN and CUR) and feedback conditions (CFB and DFB) on indices of brain activation (mean oxy‐Hb changes and relative changes in beta power) and task performance (movement errors and velocities) for each ROI. Moreover, the indices of brain activation differed significantly between the feedback conditions, and follow‐up analyses with paired *t*‐tests were conducted to assess the differences between DFB and CFB for each type of reaching task. We also used a nonparametric Wilcoxon signed‐rank test to assess the differences in agency scores between DFB and CFB for each type of reaching task. The Bonferroni correction was applied to adjust the significance level for multiple tests (*α* = 0.0125). The statistical package for the social sciences (SPSS, Version 19.0, IBM Co. Ltd., USA) was used for statistical analysis. Statistical significance was set at *p* < .05.

## 3 RESULTS

### 3.1 Behavioral results

**FIGURE 4:**
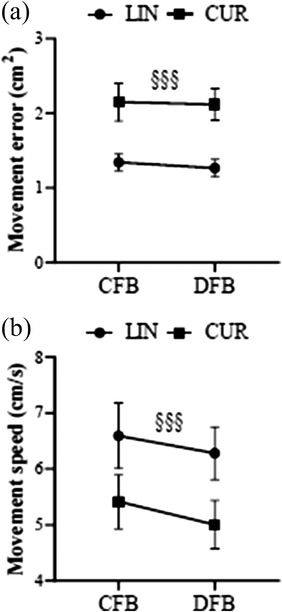
Comparisons of mean values of behavioral measures. Comparisons of the mean values for movement error (a) and movement velocity (b) between the experimental conditions. The symbol “§” indicates the significant main effects for the reaching task. Data are expressed as the means ± SEs. Abbreviations: CUR, curved reaching task; CFB, coupled feedback condition; DFB, decoupled feedback condition; LIN, linear reaching task

Two‐way repeated‐measures ANOVA revealed the main effects of reaching tasks on the movement error (*F*_1,15_ = 19.667, *p* < .001) and velocity (*F*_1,15_ = 27.917, *p* < .001) (Figure 4), with a greater error and slower velocity during CUR than LIN. The Wilcoxon signed‐rank test revealed that agency judgments in CUR were significantly lower during DFB than during CFB (*p* = .011, survived after Bonferroni correction), whereas no difference in agency experience with LIN was found between the feedback conditions.

### 3.2 fNIRS results

**FIGURE 5:**
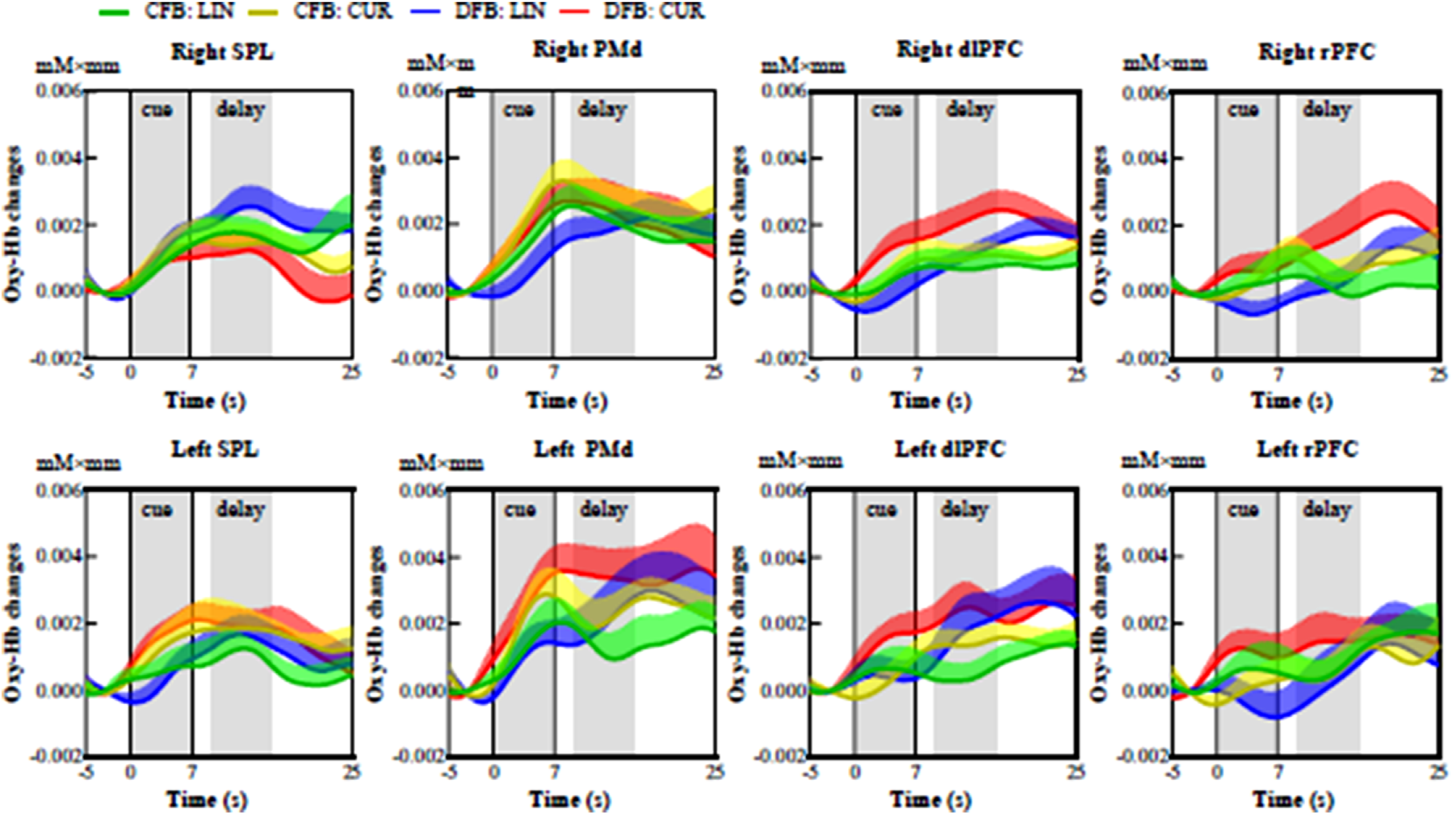
Grand‐average region of interest (ROI) time courses of oxygenated hemoglobin (oxy‐Hb) responses during the planning period. Grand‐average ROI time courses of oxy‐Hb responses during the planning period. The gray areas highlight the periods of task planning phases (cue and delay planning phases) defined for the analysis. Data are expressed as the means ± standard errors. Abbreviations: CFB, coupled feedback condition; CUR, curved reaching task; DFB, decoupled feedback condition; dlPFC, dorsolateral prefrontal cortex; LIN, linear reaching task; PMd, dorsal premotor cortex; rPFC, rostral prefrontal cortex; SPL, superior parietal lobule

Figure 5 shows the grand‐average ROI time courses of oxy‐Hb responses during the planning periods. Notably, hemodynamic responses in SPL and PMd channels exhibit an increasing trend shortly after the onset of the cue period but vary widely among conditions during the delay period. This hemodynamic response pattern may indicate the significant role of SPL and PMd in reach planning, especially in the early planning phase, under various reach type and feedback conditions. Moreover, left SPL and bilateral PMd channels exhibit higher cue‐period hemodynamic changes in CUR than they do in LIN. Regarding the prefrontal cortical (PFC) channels, while no obvious hemodynamic changes during both cue and delay periods were revealed in CFB, oxy‐Hb changes in DFB were higher during the delay period than during the cue period.

**FIGURE 6:**
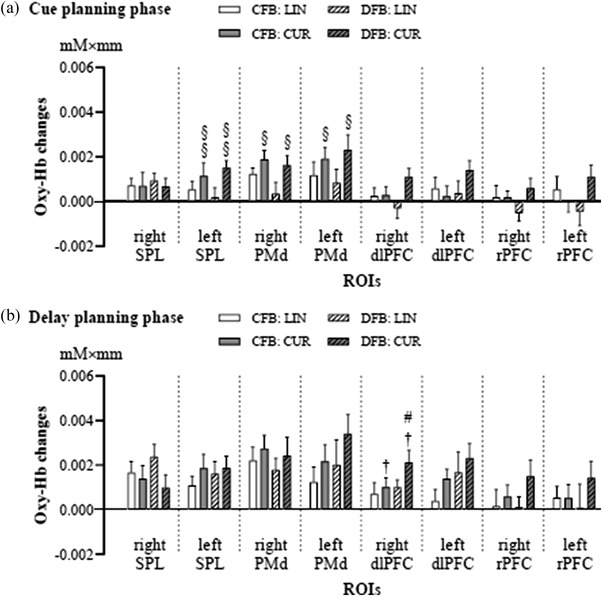
Comparisons of the average region of interest (ROI) change in oxygenated hemoglobin (oxy‐Hb) concentration. Comparisons of the average ROI change in oxy‐Hb concentration for the (a) cue planning phase and (b) delay planning phase. The symbol “#” designates the significant planned difference between the two feedback conditions for the relevant specific reaching task. Data are expressed as the means ± standard errors. The symbols “§” and “†” indicate the significant main effects for the reaching task and feedback condition, respectively. Abbreviations: CFB, coupled feedback condition; CUR, curved reaching task; DFB, decoupled feedback condition; dlPFC, dorsolateral prefrontal cortex; LIN, linear reaching task; PMd, dorsal premotor cortex; rPFC, rostral prefrontal cortex; SPL, superior parietal lobule

Regarding the cue planning phase (Figure 6a), two‐way repeated‐measures ANOVAs revealed main effects of reaching task on the oxy‐Hb response in the left SPL (*F*_1,15_ = 9.192, *p* = .008) and the left (*F*_1,15_ = 6.976, *p* = .019) and right (*F*_1,15_ = 5.314, *p* = .036) PMd, with higher oxy‐Hb responses in the left SPL and bilateral PMd during CUR than LIN.

Regarding the delay planning phase (Figure 6b), two‐way repeated‐measures ANOVAs revealed a main effect of feedback condition on the oxy‐Hb response in the right dlPFC (*F*_1,15_ = 5.356, *p* = .035), with a greater oxy‐Hb response in the right dlPFC during DFB than CFB. Furthermore, follow‐up analyses showed significantly higher oxy‐Hb responses during DFB than CFB in CUR (*p* = .011, survived after Bonferroni correction) but not LIN.

Accordingly, we also performed hemodynamic analyses on the fNIRS data related to the execution period (Figures S1 and S2). During the execution period (Figure S1), hemodynamic responses in SPL and PMd channels rise rapidly and peak a few seconds after the onset of action. These changes seem indifferent among conditions. PFC channels, unlike SPL and PMd channels, exhibit relatively slight hemodynamic changes. These hemodynamic profiles may indicate the involvement of the frontoparietal regions, mainly the SPL and PMd, in performing reaching movements under various reach type and feedback conditions. Regarding the execution phase (Figure S2), two‐way repeated‐measures ANOVAs found neither main effect of feedback condition nor reaching task on the oxy‐Hb response.

### 3.3 EEG results

**FIGURE 7:**
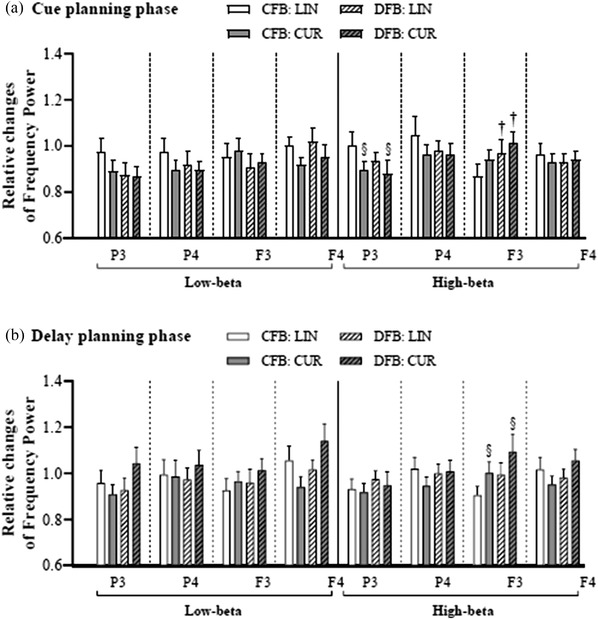
Comparisons of the channel‐related relative change in low‐beta and high‐beta powers. Comparisons of the channel‐related relative change in low‐ and high‐beta powers for the (a) cue planning phase and (b) delay planning phase. The symbols “§” and “†” indicate the significant main effects for the reaching task and feedback condition, respectively. Data are expressed as the means ± standard errors. Abbreviations: CFB, coupled feedback condition; CUR, curved reaching task; DFB, decoupled feedback condition; LIN, linear reaching task

Regarding the cue planning phase (Figure 7a), two‐way repeated‐measures ANOVAs revealed a main effect of the reaching task on the high‐beta power at P3 (*F*_1,15_ = 5.890, *p* = .028) and a main effect of the feedback condition on the high‐beta power at F3 (F_1,15_ = 5.696, *p* = .031), with a stronger high‐beta power decrease at P3 during CUR than LIN and a greater high‐beta power increase at F3 during DFB than CFB.

Regarding the delay planning phase (Figure 7b), two‐way repeated‐measures ANOVAs showed a main effect of the reaching task on the high‐beta power at F3 (*F*_1,15_ = 4.782, *p* = .045), with greater high‐beta power at F3 during CUR than LIN.

## 4 DISCUSSION

In this study, we aimed to examine the involvement of human frontoparietal regions in curved reach planning under coupled and decoupled visuomotor feedback conditions, as measured by simultaneous fNIRS‐EEG. Our results revealed that the frontoparietal regions were involved in both planning and executing the reaching movements under various reach type and feedback conditions. While there was no significant difference in brain activity among conditions during the execution period, hemodynamic responses in the SPL, PMd, and dlPFC changed significantly during the reach planning periods and were modulated differentially across both reaching tasks and feedback conditions. CUR showed higher cue‐period hemodynamic responses in the left SPL and bilateral PMd than LIN. Moreover, our EEG analyses revealed that the high‐beta power at P3 was decreased with CUR relative to LIN during the cue period. Notably, for the contrast between DFB and CFB, increased hemodynamic changes in the right dlPFC were observed during the delay period. Our subsequent analysis indicated that this greater activation of the right dlPFC was observed exclusively in CUR but not in LIN. According to our behavioral data, CUR was more spatially and kinematically complex than LIN. Although there was no difference in behavior parameters between the two feedback conditions, we found a decrease in the sense of agency in CUR but not in LIN when comparing the DFB and CFB.

### 4.1 Frontoparietal involvement in reach planning of CUR versus LIN

In line with our hypotheses, the comparison of CUR versus LIN revealed increased cue‐period hemodynamic responses in the left SPL and bilateral PMd. In this study, two reach planning tasks with the same set of starting and target points but different instructed reach trajectories were compared, allowing an assessment of the changes in brain activity associated with trajectory‐path processing. Due to the unconstrained setting, our study also provided a more robust reproduction of the brain activity underlying the naturalistic reaching function than previous fMRI studies. The neuromechanical differences between these two reaching tasks were evaluated under both coupled and decoupled feedback conditions, leading to better validation of the results. Therefore, our findings suggest that, during planning curved reaching movements, the engagement of both SPL and PMd activities—though not individually—plays a critical role in encoding the computationally demanding aspects of trajectory‐path representations.

The role of SPL regions in visuomotor transformation for curved reaching movements has been independently reported in several nonhuman primate studies (Hauschild et al., 2012; Torres et al., 2013). Cue‐period activity in the SPL may be associated with encoding upcoming reach trajectories in spatial terms (Pilacinski et al., 2018). The SPL neurons represent future curved reaching movements as spatial (Hauschild et al., 2012) and biomechanical components of impending reaching trajectories (Torres et al., 2013). Moreover, our neurophysiological data showed a decreased high‐beta power observed in the left parietal cortex during curved reach planning relative to linear reach planning. Converging evidence has indicated that beta activity during motor planning may be linked to the processing of future movement trajectories. For example, previous brain‐computer interface (BCI) research has reported that the trajectory‐related parameters of imagined upper limb movements can be decoded from beta oscillations in the brain (Korik et al., 2018). Parietal beta oscillations have also been found to represent sensorimotor integration (Donner & Siegel, 2011; Hipp et al., 2011), which is crucial for the early planning processing of target‐directed reaches (Sober & Sabes, 2005). The decreased beta power in parietal regions was reported to reflect the processing of the visuomotor transformation for future arm movements (Perfetti et al., 2011; Tombini et al., 2009). Thus, the results of this study, together with those of earlier studies, suggest the importance of SPL activity for trajectory‐path processing of forthcoming curved reaching movements.

Our hemodynamic findings also indicate the involvement of bilateral PMd in planning curved reaching movements. A bilateral increase in premotor cortical activation has consistently been observed during visuomotor task planning (Beurze et al., 2010; Hoshi, 2013). Furthermore, PMd activity synergizes with SPL activity in the initial planning processes that encode the neuronal representation of future reaching movements (Pilacinski et al., 2018). Evidence in humans and nonhuman primates also suggests that damage to PMd regions impairs the ability to perform actions along complex or curved trajectories (Rosene & Hoesen, 1977; Wong et al., 2019). Based on a previous fMRI study of curved reach planning with the same initial and target positions but varied trajectory paths, the authors suggested that PMd activity represents essential properties of future curved reach trajectories (Pilacinski & Lindner, 2019). Overall, our results—combined with previous evidence—support the idea that both the PMd and SPL play crucial roles in processing trajectory‐path representations for upcoming curved reaching movements.

### 4.2 Increased planning activity in the dlPFC of DFB versus CFB

In this study, DFB showed a higher activation in the right dlPFC during the delay period relative to CFB. This finding supports a previous study which reported that extensive engagement under decoupled feedback conditions modulates the premovement activity of the right dlPFC associated with complex visuomotor tasks (Granek et al., 2010). The involvement of the right dlPFC in preparing action plans for visuomotor tasks has been consistently reported (Goto et al., 2011; Hoshi, 2013). Numerous studies have indicated that dlPFC delay‐period activity subserves top‐down control over downstream regions to drive motor planning through visuomotor rules (Amemori & Sawaguchi, 2006; Hoshi, 2013; Tanji et al., 2007). Furthermore, the dlPFC has anatomical connections with other frontoparietal cortical regions (Miller & Cohen, 2001; Petrides, 2005), with each of these regions encoding spatial information embedded in distinct reference frames (Andersen & Buneo, 2002; Beurze et al., 2010). These connections enable the dlPFC to evaluate multidimensional spatial information, which is necessary for the coordinate processing that transforms reaching plans from eye to hand‐centered coordinates when visual and somatosensory inputs from hand motions are spatially dissociated. Earlier studies have also shown that dlPFC activity becomes functionally coordinated with other frontoparietal processes if reaching plans require the integration of visuomotor rules that govern eye‐hand coordination (Abe & Hanakawa, 2009; Hoshi, 2013). Therefore, these data suggest that the DLPFC is vital for planning reaching movements under decoupled feedback conditions and may serve as an essential part of a neuronal network that represents relevant transformational rules and incorporates them into reaching plans.

When comparing DFB versus CFB, our results revealed that an increased delay‐period activity in the right dlPFC was observed in CUR but not in LIN. In DFB, straight or linear reaching movements may involve simple transformational rules for visuomotor transformation (e.g., dragging the stylus straight ahead to move the cursor upward) (Granek & Sergio, 2015). Meanwhile, such transformational rules and the process of integrating them into reaching plans are seemingly more computationally demanding for complex or curved reaching movements. Similarly, when comparing DFB and CFB, the analysis of agency experience showed a decrease in agency judgments for CUR but not for LIN. This indicates that, during DFB, the spatial coupling between visual and somatosensory senses of hand positioning is more pronounced when movements are curved versus linear. These movements might engage extra computational processing from the dlPFC for visuomotor transformation. Although we found no significant interaction with dlPFC activity, possibly due to the limited sample size, our observations suggest that the dlPFC may be important for planning curved reaching movements under decoupled feedback conditions. However, future studies with larger sample sizes are needed to verify and extend our results.

Unlike earlier studies, we did not observe any differences in the planning activities of the SPL or PMd between the feedback conditions. One possible explanation for this discrepancy comes from a series of studies conducted in nonhuman primates by Sayegh et al. wherein subregions within the SPL and PMd appeared to function differently under distinct feedback conditions (Sayegh et al., 2013, 2014). Accordingly, reach planning under coupled feedback conditions seems to primarily engage the more caudal part of the PMd and SPL subregions adjacent to the medial intraparietal sulcus. Conversely, rostral PMd and caudal SPL processes are highly recruited during reach planning under decoupled feedback conditions. These findings suggest that functional neuroimaging of fine‐scale subregions in the SPL and PMd will be better at detecting changes in neural activity under different feedback conditions. Another explanation may be interindividual variability in participants’ experiences with different feedback conditions, which this study did not assess in detail. Extensive engagement with decoupled feedback conditions can demonstrably shape the frontoparietal activities involved in planning visually guided movements (Granek et al., 2010). Thus, the present study revealed no differences in large‐scale planning activities in the SPL and PMd between different feedback conditions. Future studies could focus on the role of subdivisions within human frontoparietal regions and control for the effects of an individual's experience with decoupled feedback conditions.

### 4.3 Implications and future directions

The present study, which employed a more realistic experimental setting than previous studies, shows that the synergy between SPL and PMd activities, rather than either independently, play major roles in processing trajectory‐path representations during curved reach planning. Our findings facilitate a deeper grasp on how the human brain reacts when prospective movements are planned along curved trajectories, which has not been studied extensively. This knowledge is needed for comprehending the cortical effects of arm reaching practices, further aiding in the improvement of rehabilitation strategies and interventions in patients with frontoparietal cortex damage or reduced reaching ability. Future research with increased spatial resolution and improved design, such as adopting connectivity analysis, is needed to corroborate our findings and extend the involvement of the frontoparietal network in complex reach planning.

Our results also suggest that planning reaching movements—particularly curved reaches—in decoupled feedback conditions may require additional frontoparietal processes, with the dlPFC actively participating, that address the separation between visual and somatosensory senses of hand positioning. As technology evolves, sensorimotor training in virtual feedback conditions has become more commonly applied in rehabilitation (Crocher et al., 2019). Our findings help supplement the underlying neural mechanisms of therapeutic training with decoupled feedback, allowing a better understanding of its efficacy on upper‐limb rehabilitation. Our study may provide a useful baseline for future studies further investigating the neural correlates of motor planning under decoupled feedback conditions in healthy and clinical populations. Furthermore, brain activity during motor planning or motor imagery has been employed in previous BCI systems to decode impending reaching movements to assist patients with impaired motor function via external devices (e.g., assistive exoskeletons, neuroprosthetics) (Kim et al., 2015; Roy et al., 2016). Attempts in this field are encouraged to incorporate signals from trajectory‐related frontoparietal regions, such as the SPL, PMd, and dlPFC, to enhance BCI performance.

This study also demonstrates the capability of combined fNIRS with EEG to investigate the changes in cortical activity during motor planning in unrestricted settings, supporting the use of concurrent fNIRS and EEG recordings in further research on realistic visuomotor tasks.

### 4.4 Limitations

First, despite the benefits of simultaneous fNIRS and EEG recording, the spatial resolution of this study was suboptimal for imaging fine‐scale cortical subregions. Future neuroimaging studies with greater spatial resolution (e.g., high‐density fNIRS‐EEG recordings) are needed to acquire more detailed data on cortical activity. The second limitation is that our study only investigated task‐related regional cortical activities, which may have overlooked the complicated functional interactions within the frontoparietal network. The coupling of regional cortical processes and functional connectivity can aid in providing comprehensive knowledge of how the brain functions during curved reach planning. Additional research in this field is recommended to measure functional frontoparietal connectivity to extend our results. Finally, as this is the first study to investigate the neural mechanisms underlying curved reach planning under various feedback conditions, several design aspects can be improved on in future research, such as including a larger number of trials or additional fitting interval settings for task periods.

## 5 CONCLUSION

In this study, using simultaneous fNIRS and EEG recordings and an unconstrained setting, we investigated frontoparietal activity during curved reach planning in both decoupled and coupled feedback conditions. Our results suggest that when movements are planned along curved trajectories, both the SPL and PMd regions exhibit crucial involvement in trajectory‐path processing. Moreover, planning reaching movements, especially curved reaches, under decoupled feedback may involve extra frontoparietal processes, with the dlPFC playing a significant role, to consider the vision‐proprioception dissociation of hand positioning. These findings will aid in enhancing the existing understanding of the neural mechanisms underlying curved reach planning. This knowledge is necessary for comprehending the brain effects of arm reaching exercises under different feedback conditions, further facilitating the advancement of rehabilitation tactics and therapies in populations with frontoparietal cortex injuries or impaired reach function.

## CONFLICT OF INTEREST

The authors declare no conflict of interest.

## AUTHOR CONTRIBUTIONS

Duc Trung Le, Hiroki Ogawa, Masato Tsuyuhara, Kazuki Watanabe, and Susumu Urakawa performed the experiments. Tatsunori Watanabe, Ryosuke Ochi, Hisao Nishijo, and Masahito Mihara technically supported the experiments. Tatsunori Watanabe, Hisao Nishijo, Naoto Fujita, and Susumu Urakawa interpreted the results of the experiments. Duc Trung Le and Susumu Urakawa prepared the figures and tables. Duc Trung Le, Tatsunori Watanabe, and Susumu Urakawa drafted the manuscript. Duc Trung Le, Tatsunori Watanabe, Ryosuke Ochi, Hisao Nishijo, Masahito Mihara, Naoto Fujita, and Susumu Urakawa approved the final version of the manuscript. Duc Trung Le, Tatsunori Watanabe, Naoto Fujita, and Susumu Urakawa edited and revised the manuscript.

## Supporting information

Figure S1 Grand average ROI time courses of oxy‐Hb responses during the execution period. Grand‐average ROI time courses of oxy‐Hb responses during the execution period. The gray areas highlight the periods of task execution phases defined for the analysis. Oxy‐Hb, oxy‐hemoglobin; LIN, linear reaching task; CUR, curved reaching task; CFB, coupled feedback condition; DFB, decoupled feedback condition; SPL, superior parietal lobule; PMd, dorsal premotor cortex; dlPFC, dorsolateral prefrontal cortex; rPFC, rostral prefrontal cortex. Oxy‐Hb, oxy‐hemoglobin; SPL, superior parietal lobule; PMd, dorsal premotor cortex; dlPFC, dorsolateral prefrontal cortex; rPFC, rostral prefrontal cortex. Data are expressed as means ± standard errors (SEs).
Figure S2: Comparisons of the average ROI change in oxy‐Hb concentration during the execution period. Comparisons of the average ROI change in oxy‐Hb concentration for execution phase. Oxy‐Hb, oxy‐hemoglobin; LIN, linear reaching task; CUR, curved reaching task; CFB, coupled feedback condition; DFB, decoupled feedback condition; SPL, superior parietal lobule; PMd, dorsal premotor cortex; dlPFC, dorsolateral prefrontal cortex; rPFC, rostral prefrontal cortex. Data are expressed as means ± standard errors (SEs).
Table S1 Results of average changes in deoxy‐Hb concentration by conditions for three tasks phases. The results were multiplied by 1000 for reporting purposes. ROI, region of interest; SD, standard deviation; LIN, linear reaching task; CUR, curved reaching task; CFB, coupled feedback condition; DFB, decoupled feedback condition; SPL, superior parietal lobule; PMd, dorsal premotor cortex; dlPFC, dorsolateral prefrontal cortex; rPFC, rostral prefrontal cortex.
Table S2 Results of two‐way repeated‐measures analysis of variance (ANOVA) on average deoxy‐Hb concentration changes by conditions for three task phases. ROI, region of interest; F, F‐measure; LIN, linear reaching task; CUR, curved reaching task; CFB, coupled feedback condition; DFB, decoupled feedback condition; SPL, superior parietal lobule; PMd, dorsal premotor cortex; dlPFC, dorsolateral prefrontal cortex; rPFC, rostral prefrontal cortex. *p < .05, **p < .01.

## Data Availability

The datasets analyzed during the current study are available from the corresponding author upon reasonable request.
