# Supplementary material for: Coupled versus decoupled visuomotor feedback: Differential frontoparietal activity during curved reach planning on simultaneous functional near‐infrared spectroscopy and electroencephalography
Source: Brain Behav. 2022 Jun 14;12(7):e2681. doi: 10.1002/brb3.2681 (PMC9304848; doi:10.1002/brb3.2681)
Supplement: Supplementary file 1 — Figure S1 Grand average ROI time courses of oxy‐Hb responses during the execution period. Grand‐average ROI time courses of oxy‐Hb responses during the execution period. The gray areas highlight the periods of task execution phases defined for the analysis. Oxy‐Hb, oxy‐hemoglobin; LIN, linear reaching task; CUR, curved reaching task; CFB, coupled feedback condition; DFB, decoupled feedback condition; SPL, superior parietal lobule; PMd, dorsal premotor cortex; dlPFC, dorsolateral prefrontal cortex; rPFC, rostral prefrontal cortex. Oxy‐Hb, oxy‐hemoglobin; SPL, superior parietal lobule; PMd, dorsal premotor cortex; dlPFC, dorsolateral prefrontal cortex; rPFC, rostral prefrontal cortex. Data are expressed as means ± standard errors (SEs). Figure S2: Comparisons of the average ROI change in oxy‐Hb concentration during the execution period. Comparisons of the average ROI change in oxy‐Hb concentration for execution phase. Oxy‐Hb, oxy‐hemoglobin; LIN, linear reaching task; CUR, curved reaching task; CFB, coupled feedback condition; DFB, decoupled feedback condition; SPL, superior parietal lobule; PMd, dorsal premotor cortex; dlPFC, dorsolateral prefrontal cortex; rPFC, rostral prefrontal cortex. Data are expressed as means ± standard errors (SEs). Table S1 Results of average changes in deoxy‐Hb concentration by conditions for three tasks phases. The results were multiplied by 1000 for reporting purposes. ROI, region of interest; SD, standard deviation; LIN, linear reaching task; CUR, curved reaching task; CFB, coupled feedback condition; DFB, decoupled feedback condition; SPL, superior parietal lobule; PMd, dorsal premotor cortex; dlPFC, dorsolateral prefrontal cortex; rPFC, rostral prefrontal cortex. Table S2 Results of two‐way repeated‐measures analysis of variance (ANOVA) on average deoxy‐Hb concentration changes by conditions for three task phases. ROI, region of interest; F, F‐measure; LIN, linear reaching task; CUR, curved reaching task; CFB, coupled feedback conditi [file BRB3-12-e2681-s001.docx]

**Figure S1:** Grand average ROI time courses of oxy-Hb responses during the execution period.

**
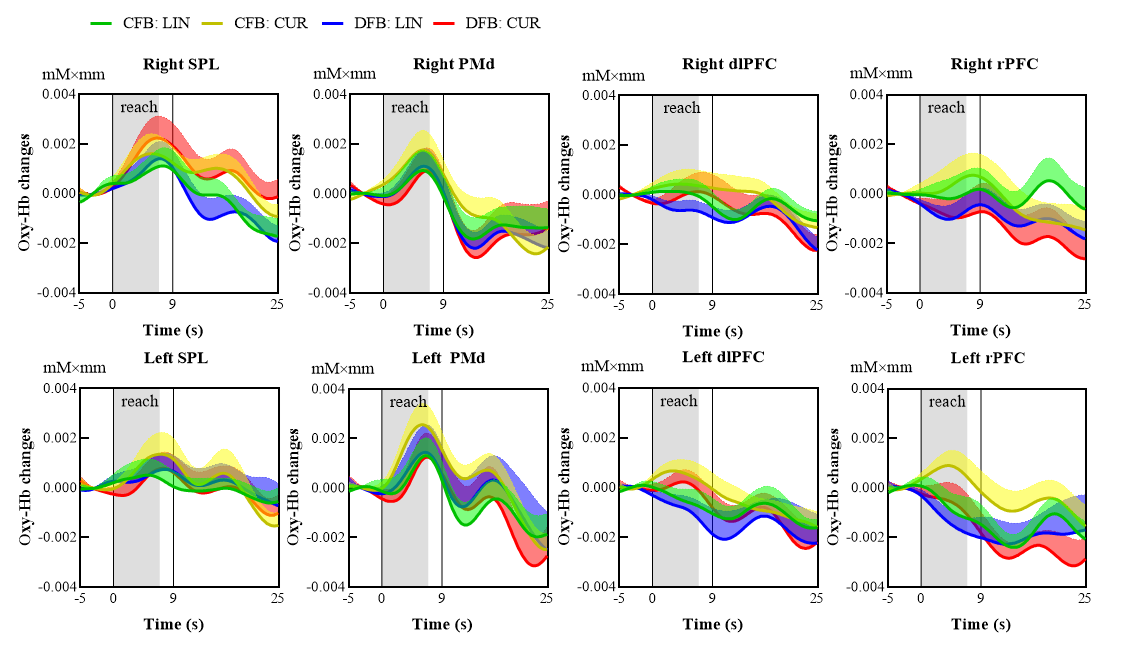
**Grand-average ROI time courses of oxy-Hb responses during the execution period. The gray areas highlight the periods of task execution phases defined for the analysis. Oxy-Hb, oxy-hemoglobin; LIN, linear reaching task; CUR, curved reaching task; CFB, coupled feedback condition; DFB, decoupled feedback condition; SPL, superior parietal lobule; PMd, dorsal premotor cortex; dlPFC, dorsolateral prefrontal cortex; rPFC, rostral prefrontal cortex. Oxy-Hb, oxy-hemoglobin; SPL, superior parietal lobule; PMd, dorsal premotor cortex; dlPFC, dorsolateral prefrontal cortex; rPFC, rostral prefrontal cortex. Data are expressed as means ± standard errors (SEs).

**Figure S2:** Comparisons of the average ROI change in oxy-Hb concentration during the execution period.

Comparisons of the average ROI change in oxy-Hb concentration for execution phase. Oxy-Hb, oxy-hemoglobin; LIN, linear reaching task; CUR, curved reaching task; CFB, coupled feedback condition; DFB, decoupled feedback condition; SPL, superior parietal lobule; PMd, dorsal premotor cortex; dlPFC, dorsolateral prefrontal cortex; rPFC, rostral prefrontal cortex. Data are expressed as means ± standard errors (SEs).

**Table S1:**  Results of average changes in deoxy-Hb concentration by conditions for three tasks phases. The results were multiplied by 1000 for reporting purposes. ROI, region of interest; SD, standard deviation; LIN, linear reaching task; CUR, curved reaching task; CFB, coupled feedback condition; DFB, decoupled feedback condition; SPL, superior parietal lobule; PMd, dorsal premotor cortex; dlPFC, dorsolateral prefrontal cortex; rPFC, rostral prefrontal cortex.

| **Cue planning phase** | | | | | | | | |
| --- | --- | --- | --- | --- | --- | --- | --- | --- |
| **ROI** | **CFB** | | | | **DFB** | | | |
|  | **LIN** | | **CUR** | | **LIN** | | **CUR** | |
|  | **Mean** | **SD** | **Mean** | **SD** | **Mean** | **SD** | **Mean** | **SD** |
| **Right SPL** | -0.023 | 0.923 | 0.467 | 0.994 | -0.152 | 1.033 | 0.572 | 0.902 |
| **Left SPL** | 0.044 | 1.619 | 0.160 | 1.457 | 0.220 | 1.440 | -0.154 | 1.170 |
| **Right PMd** | -0.018 | 1.083 | -0.171 | 1.205 | 1.232 | 1.122 | 0.718 | 1.086 |
| **Left PMd** | 0.166 | 0.879 | 0.100 | 1.311 | 0.897 | 2.268 | 0.136 | 1.647 |
| **Right dlPFC** | 0.245 | 0.751 | 0.595 | 1.010 | 0.567 | 1.029 | -0.069 | 0.823 |
| **Left dlPFC** | 0.018 | 0.867 | 0.444 | 0.961 | 0.303 | 1.141 | -0.146 | 1.477 |
| **Right rPFC** | 0.099 | 0.968 | 0.336 | 0.992 | 0.369 | 0.964 | 0.000 | 0.948 |
| **Left rPFC** | 0.160 | 1.095 | 0.696 | 1.128 | 0.086 | 1.841 | -0.225 | 1.931 |
| **Delay planning phase** | | | | | | | | |
| **ROI** | **CFB** | | | | **DFB** | | | |
|  | **LIN** | | **CUR** | | **LIN** | | **CUR** | |
|  | **Mean** | **SD** | **Mean** | **SD** | **Mean** | **SD** | **Mean** | **SD** |
| **Right SPL** | -0.147 | 1.265 | 0.667 | 1.741 | -0.537 | 1.618 | 0.016 | 1.128 |
| **Left SPL** | -0.089 | 2.117 | 0.265 | 2.316 | -0.027 | 1.350 | -0.419 | 1.822 |
| **Right PMd** | -1.244 | 1.937 | -0.576 | 1.816 | 0.435 | 1.475 | -0.623 | 1.686 |
| **Left PMd** | 0.079 | 2.761 | 0.081 | 2.042 | -0.003 | 3.645 | -0.162 | 2.301 |
| **Right dlPFC** | -0.545 | 1.451 | -0.126 | 1.832 | -0.021 | 0.682 | -0.623 | 1.770 |
| **Left dlPFC** | 0.036 | 1.457 | -0.313 | 1.572 | -1.053 | 2.832 | -0.552 | 1.477 |
| **Right rPFC** | -0.764 | 1.851 | 0.071 | 1.714 | -0.262 | 1.472 | -0.620 | 1.978 |
| **Left rPFC** | 0.219 | 1.827 | 0.820 | 1.725 | -1.069 | 2.868 | -0.352 | 2.489 |
| **Execution phase** | | | | | | | | |
| **ROI** | **CFB** | | | | **DFB** | | | |
|  | **LIN** | | **CUR** | | **LIN** | | **CUR** | |
|  | **Mean** | **SD** | **Mean** | **SD** | **Mean** | **SD** | **Mean** | **SD** |
| **Right SPL** | -0.254 | 1.195 | -0.061 | 1.046 | -0.027 | 0.833 | -0.036 | 1.045 |
| **Left SPL** | 0.084 | 1.672 | -0.388 | 1.618 | 0.060 | 1.390 | 0.288 | 1.275 |
| **Right PMd** | -0.187 | 1.537 | -0.825 | 1.500 | 0.074 | 1.053 | -0.341 | 1.331 |
| **Left PMd** | -0.336 | 1.502 | -1.005 | 2.414 | -0.169 | 1.828 | -1.215 | 2.100 |
| **Right dlPFC** | -0.322 | 0.870 | -0.405 | 1.266 | 0.232 | 0.760 | 0.036 | 0.754 |
| **Left dlPFC** | 0.237 | 0.921 | -0.290 | 1.581 | 0.490 | 1.019 | 0.020 | 0.832 |
| **Right rPFC** | -0.246 | 0.754 | -0.759 | 1.370 | -0.277 | 1.075 | 0.293 | 1.422 |
| **Left rPFC** | 0.342 | 1.040 | -0.519 | 1.476 | 0.231 | 0.938 | 0.054 | 1.648 |

**Table S2:** Results of two-way repeated-measures analysis of variance (ANOVA) on average deoxy-Hb concentration changes by conditions for three task phases. ROI, region of interest; F, F-measure; LIN, linear reaching task; CUR, curved reaching task; CFB, coupled feedback condition; DFB, decoupled feedback condition; SPL, superior parietal lobule; PMd, dorsal premotor cortex; dlPFC, dorsolateral prefrontal cortex; rPFC, rostral prefrontal cortex. **p* < 0.05, ***p* < 0.01.

| **Cue planning phase** | | | | | | |
| --- | --- | --- | --- | --- | --- | --- |
| **ROI** | **Feedback** | | **Reach type** | | **Interaction** | |
|  | **F** | ***p*-value** | **F** | ***p*-value** | **F** | ***p*-value** |
| **Right SPL** | 0.002 | 0.966 | 9.512 | 0.008 | 0.159 | 0.696 |
| **Left SPL** | 0.057 | 0.815 | 0.176 | 0.681 | 1.192 | 0.292 |
| **Right PMd** | 14.412 | 0.002** | 1.707 | 0.211 | 0.912 | 0.355 |
| **Left PMd** | 0.71 | 0.413 | 2.727 | 0.119 | 1.34 | 0.265 |
| **Right dlPFC** | 0.549 | 0.47 | 0.313 | 0.584 | 5.772 | 0.03 |
| **Left dlPFC** | 0.195 | 0.665 | 0.004 | 0.952 | 2.456 | 0.138 |
| **Right rPFC** | 0.026 | 0.874 | 0.073 | 0.791 | 3.612 | 0.077 |
| **Left rPFC** | 1.698 | 0.212 | 0.336 | 0.571 | 1.827 | 0.197 |
| **Delay planning phase** | | | | | | |
| **ROI** | **Feedback** | | **Reach type** | | **Interaction** | |
|  | **F** | ***p*-value** | **F** | ***p*-value** | **F** | ***p*-value** |
| **Right SPL** | 1.98 | 0.18 | 5.211 | 0.037* | 0.123 | 0.731 |
| **Left SPL** | 0.338 | 0.57 | 0.003 | 0.957 | 0.939 | 0.348 |
| **Right PMd** | 10.122 | 0.006** | 0.343 | 0.567 | 6.994 | 0.018* |
| **Left PMd** | 0.059 | 0.812 | 0.041 | 0.843 | 0.015 | 0.905 |
| **Right dlPFC** | 0.002 | 0.962 | 0.088 | 0.771 | 4.801 | 0.045* |
| **Left dlPFC** | 1.476 | 0.243 | 0.038 | 0.848 | 0.801 | 0.385 |
| **Right rPFC** | 0.115 | 0.739 | 0.698 | 0.417 | 1.55 | 0.232 |
| **Left rPFC** | 5.918 | 0.028* | 2.097 | 0.168 | 0.009 | 0.927 |
| **Execution phase** | | | | | | |
| **ROI** | **Feedback** | | **Reach type** | | **Interaction** | |
|  | **F** | ***p*-value** | **F** | ***p*-value** | **F** | ***p*-value** |
| **Right SPL** | 0.255 | 0.621 | 0.32 | 0.58 | 0.204 | 0.658 |
| **Left SPL** | 0.918 | 0.353 | 0.107 | 0.748 | 1.064 | 0.319 |
| **Right PMd** | 1.157 | 0.299 | 4.549 | 0.05 | 0.135 | 0.719 |
| **Left PMd** | 0.005 | 0.945 | 3.167 | 0.095 | 0.239 | 0.632 |
| **Right dlPFC** | 3.705 | 0.073 | 0.443 | 0.516 | 0.172 | 0.685 |
| **Left dlPFC** | 0.94 | 0.348 | 6.481 | 0.022* | 0.01 | 0.921 |
| **Right rPFC** | 2.97 | 0.105 | 0.008 | 0.929 | 3.963 | 0.065 |
| **Left rPFC** | 0.502 | 0.489 | 2.471 | 0.137 | 1.422 | 0.252 |
